# Supplementary material for: An eye for talent: The recruiters’ role in the Australian Football talent pathway
Source: PLoS One. 2020 Nov 2;15(11):e0241307. doi: 10.1371/journal.pone.0241307 (PMC7605670; doi:10.1371/journal.pone.0241307)
Supplement: S1 Appendix — (DOCX) [file pone.0241307.s001.docx]

**S1 Appendix**

Included in the appendix is the figures depicting the schematics of the second and third order themes for each primary theme.

*Appendix 1.* Schematic of the recruiter; second order themes (white boxes) and third order themes (grey boxes).

*Appendix 2.* Schematic of the processes and practice theme; second order themes (white boxes) and third order themes (grey boxes)

*Appendix 3.* Schematic of the assessment theme; second order themes (white boxes) and third order themes (grey boxes)

*Appendix 4.* Schematic of the selection theme; second order themes (white boxes) and third order themes (grey boxes)
