# Supplementary material for: An eye for talent: The recruiters’ role in the Australian Football talent pathway
Source: PLoS One. 2020 Nov 2;15(11):e0241307. doi: 10.1371/journal.pone.0241307 (PMC7605670; doi:10.1371/journal.pone.0241307)
Supplement: Attachment — Submitted filename: 09-16-20 Response to Reviewers.docx [file pone.0241307.s002.docx]

**Reviewer 1**

REVIEWER SUGGESTION 1: Overall I found the document to be very well-written, logically structured with appropriate reference to relevant literature. This is a good foundation study that will stimulate further research into best practices for recruitment in AFL.

RESPONSE – We thank the reviewer for their time and the positive feedback about the manuscript.

REVIEWER SUGGESTION 2: The discussion was thorough, yet disciplined in focusing on relevant themes only and reinforced the results. I would have liked to seen a practical applications section outlining succinctly the most common themes that dictate how recruiters decides on who they recruit to provide the reader with easy to remember take-home messages as there was a lot of information to process from this research

RESPONSE – We thank the reviewer for the positive feedback. While we are mindful of Reviewer 2’s comment in relation to the overlapping of information in the results and discussion, we have integrated the practical take-home message within the conclusion. This now states: “From a practical perspective, the findings demonstrate that recruiters consider a variety of interdependent attributes, such as technical, tactical, physiological, psychological, perceptual-cognitive and game related performance to make an informed talent identification decision. Further, these decisions are underpinned by previous recruitment decisions (either positive or negative), personal talent identification philosophy, and club philosophy and player needs.” (Page 23; Line 22 – Page 24; Line 2)

REVIEWER SUGGESTION 3: Page 26 Line 7: “addition to watching games, recruiters also have constant co and coding specific game actions”. What is constant co?

RESPONSE – We thank the reviewer for identifying this incomplete sentence within the manuscript. The sentence has not been revised to read: “In addition to watching games, recruiters code specific game actions and have regular conversations about the potential player’s performance with colleagues.” (Page 19; Lines 7 – 8)

**Reviewer 2**

REVIEWER SUGGESTION 1: I commend the authors for investigating a novel question of significance and using robust qualitative methodology. The article is well written and it is clear the authors have expertise in the area of research.

RESPONSE – We thank the reviewer for the positive comments about the manuscript.

REVIEWER SUGGESTION 2: Introduction - Line 6 consider changing to "recruitment" decisions. Additionally, do the authors have a reference to support the statement about the *ad nauseum* scrutiny of the decisions?

RESPONSE – We thank the reviewer for identifying this typographical error and have amended this in text. Further, the statement refers to anecdotal evidence from newspapers and social media, rather than empirical evidence, as such, we have modified the sentence to state “Anecdotally, recruitment decisions are retrospectively scrutinised ad nauseam by sports fans, the media and within sports organisations” (Page 2; Line 6).

REVIEWER SUGGESTION 3: In the methods section: The authors state that recruiters from 12 of the 18 clubs in the Australian Football League were interviewed. Are there multiple recruiters for each club? And what proportion of the total number of recruiters across the AFL were interviewed.

RESPONSE – We thank the reviewer for the comment, and the need to clarify participant’s club associations. To do this, we have now highlighted that there were 12 participants in total, from 12 different club. The sentence now reads: “Twelve full-time male recruiters (i.e., scouts) from 12 of the 18 AFL professional clubs participated in this study” (Page 4; Line 24). Within the AFL talent identification system, there are varying levels of employment for recruiters, from volunteer to full-time. As such, we believed it was important to consider the individuals responsible for making the final talent recruitment decision, the National Recruitment Manager. This individual is full-time at the club and responsible for making the decision for each player who is recruited to the team. In addition to interviewing 66% of the leagues National Recruitment managers, by the 12^th^ participant we were reaching data saturation.

REVIEWER SUGGESTION 4: Page 8- lines 5-6: "7" should be spelled out in full

RESPONSE – We have amended this in text (Page 8; Line 5).

REVIEWER SUGGESTION 5: Page 17 line 13: Consider changing 'tactic' to 'tactical'.

RESPONSE – The word in the sentence highlighted by the reviewer is actually tacit, relating to tacit knowledge, as such no change has been made in the document. If we however have missed the word the reviewer is highlighting then we would be happy to change.

REVIEWER SUGGESTION 6: There is a bit of overlap between the results and the discussion. Consider condensing the results section where appropriate and incorporating into the discussion. Additionally, discussing the limitations of the study within the discussion should be considered by the authors.

RESPONSE – We thank the reviewer for the comment and acknowledge the potential overlap of information between the results and discussion. We have attempted to reduce some of the information within the results section where possible. However, due to the qualitative nature of the data, we do believe some detail is required to provide context around the themes and the associated quotes in the results section, with the discussion providing the links to the research around the identified themes.

We thank to reviewer for identifying the lack of limitations within the document, and this was an oversight on our behalf. We have now included a limitations section in the manuscript (Page 23; Lines 1 – 18).
